# Surveillance of drug resistance tuberculosis based on reference laboratory data in Ethiopia

**DOI:** 10.1186/s40249-019-0554-4

**Published:** 2019-06-14

**Authors:** Getu Diriba, Abebaw Kebede, Habteyes Hailu Tola, Ayinalem Alemu, Mengistu Tadesse, Epherem Tesfaye, Zemedu Mehamed, Abiyot Meaza, Bazezew Yenew, Hilina Molalign, Biniyam Dagne, Waganeh Sinshaw, Misikir Amare, Shewki Moga, Yeshiwork Abebaw, Getachew Sied

**Affiliations:** Ethiopian Public Health Institute, Addis Ababa, Ethiopia

**Keywords:** Tuberculosis, Multidrug-resistant tuberculosis, Rifampicin resistance, Isoniazid resistance

## Abstract

**Background:**

Both passive and active surveillance of drug resistance have an important role in tuberculosis (TB) control program. Surveillance data are important to estimate the magnitude of drug resistance TB, to know the trend of the disease, assess the performance of the program, and to forecast diagnosis and treatment supplies. Therefore, this study aimed to determine the prevalence and the proportion of drug resistant tuberculosis in Ethiopia based on passively collected data.

**Methods:**

A cross-sectional study was conducted at the National Tuberculosis Reference Laboratory and seven Regional TB laboratories in Ethiopia on a retrospective data collected from July 2017 to June, 2018. Data were collected by standardized checklist from TB culture laboratory registration book. Percentage of recovery rate, contamination rate, and prevalence of drug resistance TB were determined by Statistical Package for Social Science (SPSS) version 23.

**Result:**

Of 10 134 TB suspected individuals included into this analysis, 1183 (11.7%) were culture positive. The overall contamination proportion was 5.3% and nontuberculous mycobacteria proportion was 0.98%. First-line drug susceptibility test was performed for 329 *Mycobacterium tuberculosis* complex isolates, and the proportion of resistance was 5.7 and 6.3% for isoniazid and rifampicin respectively. The proportion of multidrug-resistant tuberculosis (MDR-TB) was 4.3% in new patients, while 6.7% in previously treated patients. However, there was no category for 0.6% patients, and the overall proportion of MDR-TB was 11.6%.

**Conclusions:**

The result of this study indicated that MDR-TB is a serious public health problem in Ethiopia. Thus, strengthen prevention and control program is vital to halt the burden of drug resistant TB in the country.

**Electronic supplementary material:**

The online version of this article (10.1186/s40249-019-0554-4) contains supplementary material, which is available to authorized users.

## Multilingual abstracts

Please see Additional file 1 for translations of the abstract into the five official working languages of the United Nations.

## Background

Tuberculosis (TB) is a major global public health problem that affects millions of people across the world [1]. It is also a persistent health threat among the poorest and most vulnerable segments of the world population [1]. Drug resistant TB is the most global public health threat due to its risk to further spread across the world without boarder and its poor treatment outcome [2]. Moreover, due to increased population mobility across the globe which facilitates the continuous transmission of drug resistance TB bacilli, elimination of TB from a given country is valueless unless it eliminated globally.

About 2 billion people are infected with *Mycobacterium tuberculosis (MTB)* across the world [3], and about 10 million new active case and 1.6 million deaths occurred in 2017 alone [1]. TB remains an enormous health and economic problem not only in developing regions, but it is also the main economic problem in high-income countries due to tuberculosis/human immune deficiency virus (TB-HIV) co-infection and the emergence of multidrug resistant *MTB* strains [4].

Evidence indicated that 490 000 new cases of multidrug-resistant tuberculosis (MDR-TB) and 600 000 new cases of rifampicin-resistant Tuberculosis (RR-TB) cases are registered globally in 2017 [3]. The global drug resistance survey data shows that 30% of the 3.4 million new bacteriologically confirmed and previously treated TB cases notified globally were obtained drug susceptibility test for rifampicin [4, 5] which makes the overall service coverage of 24% for new TB patients and 53% for previously treated TB patients [4]. Although periodic active surveillance of drug resistance TB is vital and more accurate to estimate current spread of the resistance strain, it is resource intensive, required qualified manpower and financial ability. The challenges related active surveillance is the most obstacles in resource-limited countries where the burden of the disease is high. Thus, estimating the burden of the disease from the available routine laboratory diagnosis data is important to monitor the effectiveness of the program and to know the magnitude of the drug resistance in resource limited settings.

Ethiopia is one of the 30 high TB, TB/HIV and MDR-TB burdened countries [3]. It is also ranked 15th among countries with high MDR-TB countries with more than 5800 estimated MDR-TB cases each year [2, 6]. An estimated epidemiological burden of all forms of TB cases in Ethiopia is also 182 per 100 000 populations in 2017 [1]. In Ethiopia, low socioeconomic status of the population, high prevalence of infectious diseases including HIV, poor treatment outcomes, longer treatment duration, high treatment costs, and many more social and economic complications make MDR-TB a more complex disease than drug susceptible TB [7]. The overall epidemiology of drug resistant TB is not well documented in Ethiopia to support effective intervention planning due to several factors including high burden of TB/HIV co-infection (11%) [8, 9]. The recent estimate indicated that, the prevalence of MDR-TB is 2.7% in newly diagnosed cases and 14% in previously treated cases in Ethiopia [3]. The burden of MDR-TB is increasing from time to time in Ethiopia. However, there is limited evidence that indicates the prevalence of MDR-TB from routine laboratory data. In addition, as a result of lack of modern and sensitive technology in Ethiopia, MDR-TB case detection is low which could hinder the actual burden of the disease in the setup [10–12]. Although routine data severe from its poor quality, poor representativeness and incompleteness, it is important to show the current burden of the disease and to monitor the ongoing condition of the disease in resource-limited countries. Moreover, conducting passive surveillance and reporting the finding is important for annual global MDR-TB burden estimation. Therefore, this survey was aimed to estimate the prevalence of drug resistant TB in Ethiopia based on routine laboratory diagnosis data.

## Materials and methods

### Study design and area

A cross-sectional study was conducted in National Tuberculosis Reference Laboratory (NTRL) of Ethiopian Public Health Institute (EPHI) and seven regional TB culture laboratories from July 2017 to June 2018 in Ethiopia. These institutions are the only functional facilities that have TB culture and drug sensitivity testing (DST) service during the data collection time in the country. Clinical samples are referred to these laboratories from different health facilities in Ethiopia.

Ethiopia is the second populated country in Africa following Nigeria, and the government of Ethiopian has also prioritized TB control program as one of the major health problem in the country’s Health Sector Development Program [13]. The operation of the health system has been decentralized to regional governments in the country and district health offices. Most regional governments has one TB culture laboratory. Each district has a primary hospital with multiple health centers, and every health center is administratively linked to health posts. Besides, health posts are staffed with two female health extension workers who provide a package of basic services including TB prevention and treatment follow up [9].

### Sample size determination and sampling technique

All presumptive TB/MDR-TB patients who were referred to one national TB culture reference laboratory and seven regional TB culture laboratories from July 2017 to June 2018 were included consecutively from the routine laboratory registration book. Therefore, sample size was not determined because all eligible participants in the specified time period were included to the study.

### Data collection and quality assurance

Data on socio-demographic variables, culture results, and drug susceptibility test results were extracted from laboratory registration book using predesigned checklist. Regular supervision of data collection process was done by the principal investigator in all regional laboratories to assure data quality. Data collectors were trained on how the data extracted from the laboratory registration book. The collected data was checked for the consistency and completeness.

### Data analysis and interpretation

Data collected from the routine laboratory diagnosis registration book were entered into Microsoft Excel 2010, and analyzed by SPSS 23.0 (IBM Corporation, NY, Chicago). Frequencies and percentage were used to determine drug resistance level among TB patients referred to the reference laboratories in Ethiopia for TB diagnosis.

## Results

### Socio-demographic characteristics

A total of 10 134 TB cultures were performed in eight TB culture and DST laboratoires from July 2017 to June 2018. Of total TB culture performed in the specified period of time, 5502 (54.3%) were male while 4632 (45.7%) female. The majority (97.1%) of TB patients were in the age group of > 15 years. Of the total TB culture performed in the indicated laboratories 1020 (10%) were to diagnose drug resistance TB and 9114 (90%) for follow up during treatment course. Among the total culture performed in the indicated laboratories the majority (90%) were to monitor the treatment.

### Culture results

**Fig. 1:**
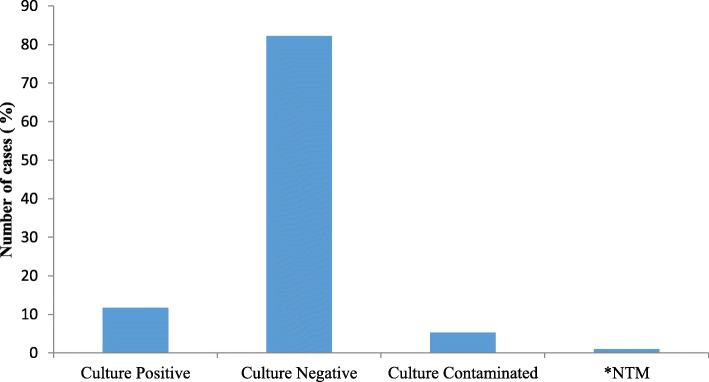
Frequency and type of culture results performed at all laboratories from July 2017 to June 2018 (*n* = 10 134). * NTM: Nontuberculous mycobacteria

Of the total culture performed 1183 (11.67%) were positive, 8322 (82.2%) negative, 99 (0.98%) nontuberculous mycobacteria (NTM) and 530 (5.3%) were contaminated (Fig. 1). Of 1183 culture positive cases, 448 (37.9%) were new, while 735 (62.1%) were on MDR-TB treatment follow up. The largest (31.6%) number of TB culture was performed in EPHI national TB reference laboratory and followed by Adama Public Health Research and Referral Laboratory Center (16.0%). The lowest number of culture was performed in Jimma University Hospital Mycobacteriology Research Center (5.8%) (Table 1).

**Table 1:** Percentage of culture performed in included laboratories from July 2017 to June 2018

Name of laboratory	Frequency (%)
Tigray Health Research Institute	932 (9.2)
Hawassa Regional Public Health Laboratory	1056 (10.4)
Harari Health Research and Regional Laboratory	771 (7.6)
Amhara Public Health Institute	1200 (11.9)
National TB Reference Laboratory, Ethiopian Public Health Institute	3199 (31.6)
Gonder University Hospital TB Culture Laboratory	765 (7.5)
Jimma University Hospital Mycobacteriology Research Center	590 (5.8)
Adama Public Health Research and Referral Laboratory Center	1621 (16.0)
Toala	10 134 (100.0)

### First line anti-tuberculosis resistance profile

Out of 329 DST performed, 243 (73.8%) were male while 86 (26.2%) female, and 322 (97.9%) were in the age group of > 15 years while 7 (2.1%) in the age group of < 15 years. Table 2 depicts the distribution of drug resistance among 329 *MTB* isolates. First line drug sensitivity test was performed for 329 *MTB* isolates. Of 329 isolates 19 (5.8%) were isoniazid (INH) mono resistant, while 22 (6.7%) were rifampicin (RIF) mono resistant. The prevalence of MDR-TB was 4.3% (14/329) in new patients and 6.7% (22/329) in previously treated patients. The overall prevalence of MDR-TB from the 329 isolates was 38 (11.6%). However, resistance category was unknown for 3.3% (11/329) patients.

**Table 2:** Drug resistance proportion to first-line anti-tuberculosis drugs from 329 patients with available DST results versus history of treatment

DST Results	New patients (*n* = 187) Frequency (%*)	Previously treated patients (*n* = 131) Frequency (%*)	Unknown patient treatment history (*n* = 11) Frequency (%*)	Total (*n* = 329) Frequency (%*)
Susceptible	149 (45.3%)	5 (1.5%)	8 (2.4%)	251 (76.3%)
Mono resistance				
INH	14 (4.3)			19 (5.8%)
RIF	10 (3.1%)	11 (3.3%)	1 (0.3%)	22 (6.7%)
MDR	14 (4.3%)	22 (6.7%)	2 (0.6%)	38 (11.6%)

%* = F× 100/*n*, *DST* Drug susceptibility testing, *RIF* Rifampicin, *INH* Isoniazid, *MDR* Multiple drug resistance

## Discussion

MDR-TB is the most serious public health problem in the world. Ethiopia is among the countries highly affected with MDR-TB. In 2017 an estimate indicated that the incidence of MDR-TB in Ethiopia is 2.7% in new cases and 14% in previously treated cases [3]. Although the burden of MDR-TB in Ethiopia is increasing, there is limited information from routine laboratory data. Thus, this study was aimed to determine the prevalence of MDR-TB in patients seeking laboratory diagnosis in reference laboratories in Ethiopia.

The routine laboratory data of 10 134 presumptive and on treatment patients were reviewed form eight reference laboratories in the country. Of 10 134 total cultures performed in the specified period of time, the overall positive culture result was 1183 (11.7%). The contamination rate of TB culture was 530 (5.23%). First line LPA susceptibility testing was performed for 329 isolates and the total isolates of 247 (75.1%) were susceptible for all drugs test done. However, 19 (5.7%) isolates were INH resistance, while 21 (6.3%) RIF resistance and 40 (12.2%) were resistant either for INH or RIF or both. The overall prevalence of MDR-TB in this survey was 38 (11.6%), and the prevalence of MDR-TB among new and previously treated patients was 4.3 and 6.7% respectively.

The overall prevalence of MDR-TB in this survey which based on the routine laboratory diagnosis data was approximately similar with previously report of national survey data [9]. Moreover, the finding of this survey was comparable with the results reported by previous studies from different parts of Ethiopia [14–16]. Our finding was also similar with the study reported from China in which the overall prevalence of MDR-TB is 10.1% [17]. Still, study reported from China indicated that similar finding with our finding [18]. In contrast, a study reported from Nigeria indicated higher (19.4%) prevalence of MDR-TB than our finding [19]. Moreover, the study reported from Saudi Arabia indicated lower prevalence of MDR-TB than our survey result [20]. The possible explanation of the difference between our finding and previous reports could be the analysis of this study based on the routine laboratory diagnosis which might not representative of the actual MDR-TB prevalence in the study setup. In other hand, this study data was collected from the laboratories where TB cases more likely diagnosed for follow up and medical attention rather than study purpose. These possible reasons might be induced the overestimation or underestimate the prevalence of MDR-TB in the study area.

In our finding the prevalence of INH resistance was 5.7% which was similar with the result reported from different African countries. For instance, the study reported from the Central African Republic indicated that the prevalence of INH resistance is 5.8% [21], and the study reported from Somalia was also shown similar figure (5.7%) [22]. Moreover, the study reported from Nigeria indicated similar prevalence (6.6%) of INH resistant with our findings [19].

The prevalence of RIF resistance was 6.3% in this study which is higher than the findings reported previously from the eastern part of Ethiopia in which the prevalence of RIF resistance was 1.9% [23]. This difference most probably related to the representativeness of the study participants and the difference in study participants. The study participants of the study reported by Seyoum et al. [23] were only new TB patients, but in our study the participants were both new and previously treated TB patients. It is clear that inclusion of previous treated TB patients can lead to high prevalence of drug resistance due to the prevalence of drug resistance is high among previously treated TB patients. However, the study reported from South Africa indicated similar burden of RIF resistance (8.8%) with our result [24].

This survey data was based on aggregate reports on manual case counts from multiple paper-based records on a quarterly basis. This might be lead to errors which could under or overestimate the prevalence of drug resistance TB in the country. In addition, this study completely based on the routine diagnosis laboratory data which collected passively. This could be compromise the representativeness of the participant which can leads to over or under estimation of the burden of drug resistance in the country. This survey data was also had several missing data on important variables which limited the assessment of factors associated with drug resistance.

## Conclusions

This study shows that the burden of MDR-TB remains a huge concern in Ethiopian. The overall prevalence of MDR-TB was 11.6% while the prevalence of INH and RIF mono resistant were 5.8 and 6.7% respectively. Strengthen drug resistance prevention and control program is vital to halt the burden of the disease in the country. In addition, comprehensive registration of routine laboratory data is important to monitor the progress of TB control program to achieve sustainable development goal that targeted TB.

## Additional file

Additional file 1: Multilingual abstracts in the five official working languages of the United Nations. (PDF 813 kb)

## Data Availability

The data is available in the hand of corresponding author and can be shared based on reasonable request.

## Abbreviations

AFB: Acid-fast Bacilli
DST: Drug sensitivity testing
EPHI: Ethiopian Public Health Institute
HIV: Human immune deficiency Virus
INH: Isoniazid
LJ: Lowenstein-Jensen
LPA: Line probe Assay
MDR: Multiple drug resistance
MGIT: Mycobacterium Growth Indicator Tube
*MTB*: *Mycobacterium tuberculosis*
MTBC: *Mycobacterium tuberculosis* complex
NTM: *nontuberculous mycobacteria*
NTRL: National tuberculosis reference laboratory
RIF: Rifampicin
RR-TB: Rifampicin resistance Tuberculosis
TB: Tuberculosis
TB/HIV: Tuberculosis/Human immune deficiency Virus
WHO: World Health Organization
ZN: Ziehl Neelsen
