# Supplementary material for: Surveillance of drug resistance tuberculosis based on reference laboratory data in Ethiopia
Source: Infect Dis Poverty. 2019 Jun 14;8:54. doi: 10.1186/s40249-019-0554-4 (PMC6567428; doi:10.1186/s40249-019-0554-4)
Supplement: Supplementary file 1 — Multilingual abstracts in the five official working languages of the United Nations. (PDF 813 kb) [file 40249_2019_554_MOESM1_ESM.pdf]

Translation of the abstract into the five official working languages of the United Nations

مراقبه مرض السل المقاوم للأدويه بناء على بيانات مختبر مرجعي فى إثيوبيا

غيتو ديريبا، أبيبا كيبيدي، هابيتيس هيلو تولا، أينااليم أليمو، مينغيستو تاديسي، إفرم تسفاي، زيمدو محمد ، أبيوت ميازا، بازايز ينيو، هيلينا مولالين، بنيام داجني، واجانيه سيناشو، ميسيكير أماري، شوكي موغا، يشوورك أبيبا ، جيتاشيو سيد

ملخص
الخلفية: تلعب كل من المراقبة السلبية والمراقبة الفعالة لمقاومة الادوية، دورًا هاما فى برنامج مكافحه مرض السل. تعد بيانات المراقبة مهمة لتقدير مدى مقاومة مرض السل للأدوية، ولمعرفة منحى تقدم المرض، وتقييم آداء البرنامج وتقدير مستلزمات التشخيص والعلاج. لذا تسعى هذه الدراسة لتحديد نسبة ومدى انتشار السل المقاوم للأدويه فى إثيوبيا بالاعتماد على تجميع سلبي للبيانات.
الأساليب: أجريت دراسة متعددة القطاعات فى المختبر المرجعي الوطني لمرض السل وسبعة مختبرات إقليمية تعنى بمرض السل فى إثيوبيا، معتمدة على بيانات مجمعة بأثر رجعي من يوليو 2017 الى يونيو 2018. تم تجميع البيانات بواسطة قائمة تدقيق موحدة من دفتر تسجيل مختبر كشف السل. تم تحديد معدل الاستشفاء، والعدوى، وانتشار مرض السل المقاوم للأدوية بواسطة برنامج إحصاء للعلوم الاجتماعيه إصدار 23.
النتائج: من بين 10134 فرد يشتبه في إصابتهم بالسل والذين شملتهم الدراسة، أثبت الفحص 1183 (11.7%) حالة موجبة. نسبة العدوى الإجمالية كانت 5.3% وكانت نسبة المتفطرات السلية غير المسببة للسل 0.98%. تم الاختبار الأوّلي للقابلية للعقارعلى 329 معقد المتفطرة السلية المعزولة لجرثومة السل، وكانت نسبة المقاومة 5.7% و 6.3% للإيزونيازيد والريفامبيسين على التوالي. لقد كانت نسبة مرض السل المقاوم لأدوية متعددة 4.3% عند المرضى الجدد بينما بلغت 6.7% عند المرضى الذين تم علاجهم سابقًا. غير أن 0.6% من المرضى لا ينتمون لأي فئة، وكانت النسبة الإجمالية لمرض السل المقاوم لأدوية متعددة 11.6%
الخاتمة: لقد بيّنت نتيجة هذه الدراسة أن مرض السل المقاوم لأدوية متعددة يشكل خطرًا يهدد الصحة العامة. وبالتالي، فإن تعزيز برنامج الوقاية والمكافحة ضروري لوقف عبء السل المقاوم للأدوية في البلاد.

Translated from English version into Arabic by Abdallah Hamdy, proofread by Karima Benkri, through

TRANSLATORS
WITHOUT BORDERS

基于埃塞俄比亚参比实验室数据监测结核病耐药性

Getu Diriba, Abebaw Kebede, Habteyes Hailu Tola, Ayinalem Alemu, Mengistu Tadesse, Epherem Tesfaye, Zemedu Mohammed, Abiyot Meaza, Bazezew Yenew, Hilina Molalign, Biniyam Dagne, Waganeh Sinshaw, Misikir Amare, Shewki Moga, Yeshiwork Abebaw, Getachew Sied

摘要
引言：耐药性的被动和主动监测在结核病控制规划中均具有重要作用。监测数据对于估计耐药性结核病的规模、了解该病的趋势、评估项目绩效以及预测诊断和治疗用品数量均具有重要意义。因此，本研究旨在通过被动收集的数据来确定埃塞俄比亚耐药性结核病的患病率和比例。
方法：收集埃塞俄比亚国家结核病参比实验室和 7 个区域结核病实验室 2017 年 7 月至 2018 年 6 月间的回顾性数据，进行横断面研究。采用标准化检查表从结核病培养实验室登记簿中收集数据。使用 SPSS 23 软件统计回收率、污染率和耐药结核病患病率。
结果：本研究纳入的 10 134 例结核疑似病例中，1183 例(11.7%)为培养阳性。总体污染比例为 5.3%，非结核分枝杆菌污染比例为 0.98%。对 329 株结核分枝杆菌进行了一线药敏试验，对异烟肼和利福平的耐药率分别为 5.7%和 6.3%。新患者中耐多药结核病(MDR-TB)的比例为 4.3%，而以前接受过治疗的患者中，这一比例为 6.7%。然而，0.6%患者无法分类，耐多药结核病的总体比例为 11.6%。
结论：本研究结果表明，耐多药结核病是埃塞俄比亚的一个严重公共卫生问题。因此，加强预防和控制项目对于遏制该国耐药结核病的负担至关重要。

Translated from English version into Chinese by Xin-Yu Feng, edited by Pin Yang

CHINESE CENTER FOR DISEASE CONTROL AND PREVENTION
NATIONAL INSTITUTE OF PARASITIC DISEASES

Surveillance de la résistance aux médicaments de la tuberculose à partir des données d'un laboratoire de référence en Éthiopie

Getu Diriba, Abebaw Kebede, Habteyes Hailu Tola, Ayinalem Alemu, Mengistu Tadesse, Epherem Tesfaye, Zemedu Mohammed, Abiyot Meaza, Bazezew Yenew, Hilina Molalign, Biniyam Dagne, Waganeh Sinshaw, Misikir Amare, Shewki Moga, Yeshiwork Abebaw, Getachew Sied

Résumé

Contexte: La surveillance passive et active de la résistance médicamenteuse joue un rôle important dans les programmes de lutte contre la tuberculose. Les données de surveillance sont cruciales pour estimer l'ampleur de la résistance de la tuberculose, connaître les tendances de la maladie, évaluer l'efficacité des programmes et prédire les besoins de diagnostic et de traitement. La présente étude avait par conséquent pour but de déterminer la prévalence et la proportion des cas de tuberculose résistante en Éthiopie, sur la base de données collectées passivement.

Méthodes: Une étude transversale rétrospective a été menée au Laboratoire de référence national sur la tuberculose et dans sept laboratoires régionaux de la tuberculose en Éthiopie, sur la base de données collectées de juillet 2017 à juin 2018. Les données ont été recueillies dans les registres de laboratoire des cultures tuberculeuses, à l'aide d'une liste de vérifications standardisée. Le pourcentage de taux de récupération, le taux de contamination et la prévalence des résistances médicamenteuses ont été déterminées à l'aide du logiciel SSPS (Statistical Package for Social Sciences), version 23.

Résultats: Sur 10 134 cas suspects de tuberculose inclus dans cette analyse, 1183 (11,7 %) étaient positifs sur cultures. Le taux global de contamination était de 5,3 % et la proportion de mycobactéries non tuberculeuses de 0,98 %. Un test de sensibilité aux médicaments de première ligne a été réalisé sur 329 isolats du complexe *Mycobacterium tuberculosis*. La proportion d'isolats résistants à l'isoniazide était de 5,7 % et celle des résistances à la rifampicine de 6,3 %. Le pourcentage de tuberculose multirésistante était de 4,3 % chez les nouveaux patients et 6,7 % chez les patients déjà traités. Toutefois, nous avons relevé 0,6 % de patients sans catégorie. La proportion totale de tuberculose multirésistante était de 11,6 %.

Conclusions: Les résultats de cette étude indiquent que la tuberculose multirésistante constitue un grave problème de santé publique en Éthiopie. Il est donc vital de renforcer les programmes de prévention et de lutte afin de mettre fin au fardeau de la tuberculose résistante dans ce pays.

Translated from English version into French by Suzanne Assenat, proofread by Laurine Crevoisier, through

TRANSLATORS
WITHOUT BORDERS

Наблюдение за туберкулезом с лекарственной устойчивостью на основании данных справочных лабораторий в Эфиопии

Гету Дириба, Абебо Кебеде, Хабтейес Хайлу Тола, Айиналем Алему, Менгисту Тадессе, Эферем Тесфайе, Земеду Мохаммед, Абийот Меаза, Базезев Йенев, Хилина Молалайн, Бинийам Дагне, Вагане Синшо, Мисикир Амаре, Шевки Мога, Йешиворк Абебо, Гетачев Сид

Реферат

Предпосылки: пассивное и активное наблюдение за лекарственной устойчивостью играет важную роль в программе профилактики туберкулёза (ТБ). Данные наблюдений играют важную роль для оценки значимости лекарственно-устойчивого ТБ, знании тенденции болезни, оценки эффективности программы и прогнозирования диагноза и доставки средств для лечения. Поэтому целью настоящего исследования является определение распространенности и пропорции заболеваемости лекарственно-устойчивым туберкулезом в Эфиопии на основе пассивно собранных данных.

Методы: перекрестное исследование было проведено в Национальной справочной лаборатории туберкулеза и семи региональных лабораториях ТБ в Эфиопии на основании ретроспективных

данных, полученных с июля 2017 г. по июнь 2018 г. Данные были собраны с помощью стандартизованного перечня из книги учета лабораторных анализов культуры ТБ. Процентное соотношение скорости выздоровления, заражения и распространенности лекарственно-устойчивого ТБ определялось с помощью «Статистического пакета для общественных наук», версия 23.
Результаты: из 10134 лиц с подозрением на ТБ, включенных в этот анализ, у 1183 (11,7%) оказался положительный анализ по культуре. Общая процентная доля заражения составила 5,3%, а доля микобактерий, не являющихся возбудителем туберкулеза — 0,98%. Тест на чувствительность к лекарственным препаратам первой линии был осуществлен в отношении 329 сложных изолятов микобактерий туберкулеза *(Mycobacterium tuberculosis)* и доля устойчивости составила 5,7% и 6,3% для изониазида и рифампицина, соответственно. Доля заболеваемости туберкулезом с множественной лекарственной устойчивостью (МЛУ ТБ) составила 4,3% у новых пациентов и 6,7% у пациентов, ранее получавших лечение. Однако, 0,6% пациентов не было отнесено в какую-либо категорию, а общая доля заболеваемости МЛУ ТБ составила 11,6%.
Выводы: результаты данного исследования свидетельствуют о том, что МЛУ-ТБ является серьезной проблемой здравоохранения Эфиопии. Поэтому усиление программы по профилактике и борьбе с ним является критическим фактором для снижения бремени от лекарственно-устойчивого ТБ в стране.

Translated from English version into Russian by Veronika Demeshchik, proofread by Alexander Somin, through

TRANSLATORS
WITHOUT BORDERS

La vigilancia de la tuberculosis resistente a los medicamentos mediante datos de referencia de laboratorios en Etiopía

Getu Diriba, Abebaw Kebede, Habteyes Hailu Tola, Ayinalem Alemu, Mengistu Tadesse, Epherem Tesfaye, Zemedu Mohammed, Abiyot Meaza, Bazezew Yenew, Hilina Molalign, Biniyam Dagne, Waganeh Sinshaw, Misikir Amare, Shewki Moga, Yeshiwork Abebaw, Getachew Sied

Resumen
Antecedentes: la vigilancia, tanto pasiva como activa, de la resistencia a los medicamentos juega un papel importante en el programa de control de la tuberculosis (TB). Los datos recopilados mediante la vigilancia son importantes para estimar la magnitud de la tuberculosis resistente a los medicamentos, conocer la tendencia de la enfermedad, evaluar el rendimiento del programa y prever suministros para el diagnóstico y tratamiento. Por consiguiente, el presente estudio tiene por objeto determinar la prevalencia y el porcentaje de tuberculosis resistente a los medicamentos en Etiopía mediante datos recopilados de manera pasiva.
Métodos: Se realizó un estudio transversal en el Laboratorio Nacional de Referencia para la Tuberculosis y en siete laboratorios regionales para la TB en Etiopía de datos retrospectivos recopilados entre julio de 2017 y junio de 2018. Los datos se recopilaron del libro de registro de cultivo de TB de los laboratorios por medio de una lista de verificación normalizada . Se determinó el porcentaje de la tasa de recuperación, la tasa de contaminación y la prevalencia de la tuberculosis resistente a los medicamentos por medio del Paquete Estadístico para las Ciencias Sociales (SPSS), versión 23.
Resultados: de las 10 134 personas con sospecha de tuberculosis incluidas en el análisis, 1 183 (11,7 %) tuvieron cultivos positivos. El porcentaje de contaminación total fue del 5,3 % y el de micobacterias tuberculosas fue del 0,98 %. Se llevó a cabo una prueba de sensibilidad a medicamentos de primera línea para 329 aislados complejos de *Mycobacterium tuberculosis*, y el porcentaje de resistencia fue del 5,7 % y del 6,3 % para la isoniazida y la rifampicina respectivamente. El porcentaje de tuberculosis multirresistente (TB-MR) fue del 4,3 % en pacientes nuevos y del 6,7 % en pacientes tratados con anterioridad. Sin embargo, no hubo ninguna categoría para el 0,6 % de los pacientes, y el porcentaje total de TB-MR fue del 11,6 %.
Conclusiones: Los resultados del presente estudio indican que la TB-MR es un problema grave de salud pública en Etiopía. Por consiguiente, es fundamental reforzar la prevención y el programa de control para detener la incidencia de la TB resistente a los medicamentos en el país.

Translated from English version into Spanish by Mayra León, proofread by Maria Paula Gorgone, through

TRANSLATORS
WITHOUT BORDERS
